# Almost Nobody Is Using ChatGPT to Write Academic Science Papers (Yet)

**DOI:** 10.3390/bdcc8100133

**Published:** 2024-10-11

**Authors:** Heather Desaire, Madeline Isom, David Hua

**Affiliations:** Department of Chemistry, University of Kansas, Lawrence, KS 66045, USA;

**Keywords:** ChatGPT, generative AI, MDPI, academic publication, AI text detection, XGBoost, ethics

## Abstract

We assessed 19,000 scientific introductions to measure the level of undisclosed use of ChatGPT in scientific papers published in 2023 and early 2024. We applied a “stylistics” approach that has previously been shown to be effective at differentiating AI-generated text from human-written text in a variety of venues. Ten different MDPI journals were selected for this study, and the rate of use of undisclosed AI writing in these journals was fairly consistent across the journals. We estimate that ChatGPT was used for writing or significant editing in about 1 to 3% of the introductions tested. This analysis is the first systematic study of detecting undisclosed ChatGPT in published manuscripts in cases where obvious indicators, such as phrases like “regenerate response”, are not present. The work demonstrates that generative AI is not polluting mainstream journals to any appreciable extent and that the overwhelming majority of scientists remain hesitant to embrace this tool for late-stage writing and editing.

## 1. Introduction

Since the introduction of ChatGPT in late 2022, many academic scientists and journal publishers have been worried about this tool’s infiltration and pollution of the scientific literature [1–3]. While some welcome its appearance and encourage its ethical use [4], over 60% of 1600 scientists recently surveyed about AI have expressed concerns about its higher propensity for increasing mistakes, misinformation, and plagiarism in the literature [5]. Journal editors were quick to implement policies that exert tight controls over authorship, a designation not allowable for AI assistants, and to require full disclosure of AI-generated (or edited) content [6,7].

Not surprisingly, though, once this enchanting tool became widely available, the robotic fingerprints of ChatGPT started appearing in articles where authors had not confessed to its usage [8]. Ethicists, serious computer scientists, and armchair tinkerers started finding papers in the literature with phrases like “regenerate response” and “as an AI language model…”, signature phrases of the world’s most popular chatbot. A highly successful sleuth who detects and publicizes these ethical *faux pas* is the French computer scientist Guillaume Cabanac [9]; his list of suspect papers is also cross-referenced on the well-known American site Retraction Watch [10]. The Cabanac/Retraction Watch list contains about 100 instances of papers where ChatGPT was most likely used, and where the authors did not disclose it originally [10].

Developing this list of “polluted literature” is a very laudable and important effort that is already generating retractions and corrections. In addition to improving the body of scientific literature as a whole, pointing out these cases is important because it turns a spotlight on instances where neither the original authors, nor the reviewers, nor the editor managed to see something that any reader who is even marginally attentive would recognize as AI wordsmanship. However, this strategy alone is insufficient to address AI incorporation into the scientific literature because it is mainly limited to finding the “obvious sightings”; it would not catch all instances of authors using ChatGPT without disclosure.

We are interested in studying, in a systematic fashion, the incorporation rate of ChatGPT into the scientific literature in cases that are more nuanced, and therefore more difficult to detect. We rationalize that most authors who decide to use AI without disclosing it have probably removed the most obvious signatures of its presence in their manuscripts. Assuming this to be true, it is conceivable that the Retraction Watch list and efforts by Cabanac and others are just the “tip of the iceberg” in terms of true AI incorporation.

But how should one find ChatGPT-generated text in these more nuanced cases? We have been developing off-the-shelf machine learning models to detect the presence of ChatGPT in the scientific literature and other domains [11,12], and the strategy we developed does not rely on phrases that are synonymous with those used by AI-based text generators. Our approach uses a variety of stylistic differences that are sometimes more prevalent in human-generated text and sometimes more prevalent in that generated by ChatGPT, and the approach can easily determine the origin of scientific text in cases where humans could not make a definitive classification, even by carefully reading the full text passages. This approach is effective for source documents from a variety of scientific journals, AI text from both GPT-3.5 and GPT-4, and cases where AI prompts were designed to obfuscate the use of AI text generators [12]. Other studies have shown that this strategy of using human-generated stylistic feature sets and supervised classification also works for distinguishing Japanese text as human or AI, for both scientific publications [13] and public comments [14]. Still others have shown that linguistic features and off-the-shelf machine learning tools can readily distinguish different AI tools from each other [15]. Finally, this stylistics method was also the cornerstone approach used to generate accurate models to distinguish student-written newspaper articles from those generated by ChatGPT at US universities [16]. While the method is now being demonstrated in multiple domains, it has not yet been used to detect ChatGPT in scientific papers “in the wild” or to measure undisclosed usage. As a result, the actual usage of undisclosed ChatGPT in the scientific literature was not known prior to the study herein.

We describe the first systematic assessment of over 19,000 scientific papers published in MDPI journals, with the goal of measuring the undisclosed, otherwise undetectable use of ChatGPT. While some have accused the publisher of unrigorous publication standards [17], there are concerns about the methods used to make this claim [18], and we note that manuscripts in MDPI journals have appeared on the infamous Retraction Watch list mentioned above at *a lower rate* than articles from certain other publishers. Furthermore, a recent independent assessment of their peer review process revealed that the MDPI system is robust and reliable [19]. We rationalized, therefore, that the MDPI editorial staff were already catching and rejecting papers (or requiring modifications) where AI usage was easy to spot but not disclosed, so articles from this publisher would be reasonably representative of a broad range of rigorously reviewed publications.

We were able to detect the undisclosed use of ChatGPT in published papers across a wide variety of journals and fields. Yet the incorporation rate was so low, estimated to be between 1 and 3%, we conclude that scientists are leveraging this tool for writing much less than we had expected. This study provides new insights, both to AI enthusiasts and ethicists, about the (slowly) evolving writing practices of academic scientists in this new age of AI.

## 2. Experimental Procedures

Assuming the use of generative AI for writing scientific papers is relatively low, a model that detects its presence must have an exquisite ability to distinguish between human-generated and AI-generated text. Consequently, the first step in this study was to develop a highly effective model for distinguishing authorship (as human or AI) in a broad selection of scientific journals. We included diverse training data. First, 1000 human-written introductions from scientific papers that were published prior to ChatGPT’s release were included from ten different journals, as shown in Table 1.

Introduction sections were the focus because other studies have shown that it is possible for this section to be written entirely by ChatGPT [20]. The ten journals used to acquire the human data in this study span diverse scientific topics, contain impact factors from 3 to 8, and are among MDPI’s fifty largest journals. See Table 1 for more details. All the human-written source documents (introduction sections of scientific papers) were written in 2022; each set of 100 documents, from each journal indicated above, was a consecutive series of 100 documents. The individual article numbers for the exact introductions used are provided in the Supplementary Materials. Next, 1000 comparator introductions from ChatGPT (GPT-3.5) were generated. Every AI comparator sample was a ChatGPT-modified version of one of the human-written documents in the training set. The prompt to ChatGPT, used to generate the AI versions of the documents, was “Use the text below to write a new introduction for a scientific paper. Your introduction should be about four paragraphs long. Do not plagiarize the original text. The numbers in brackets are references; keep only the ones in your document when you use the same information. Also, do not provide any text other than the Introduction section, and do not write Introduction at the start… [Human-written text goes here]”. Because the human-generated and AI-edited training data both originated from a fully human-written introduction, the training data from both classes were quite similar, substantially increasing the difficulty of this classification challenge.

After all the introductions were in-hand, they were automatically processed. First, every paragraph from each document in the training data was initially inserted into a single line of a data matrix that accommodated a maximum of 300 words. (Paragraphs longer than 300 words were automatically truncated). Next, the citations were deleted. In these journals, the citations appear in square brackets with no spaces between citation numbers if more than one is present, so the references were readily scrubbable in an automated fashion. Each example of square brackets, “[ ]”, appearing without spaces between the opening and closing bracket was removed, as was everything within these brackets. This step ensured there would be no detectable differences between the two types of documents based on the presence or frequency of citations. This process, performed automatically with an R script in this case, is an automated version of the process described in a previous study [11,12] where citations were removed manually by human data processors. Aside from this data-cleaning step, no other modifications were made to the documents prior to feature extraction.

Next, 23 numerical features were extracted from each paragraph, generating a 23-feature vector that represented the original paragraph. This vector was used for classifying the article type (as human or AI). The extracted features related to the articles’ stylistic differences; we [11,12,16] and others [13–15] had previously established the validity of using stylistic features to differentiate human- vs. AI-generated text in various types of writing domains [11–16]. The features used herein are optimized for this study, and all the matrices of numerical training and testing data, with 23 features per paragraph of source material, are provided in the Supplementary Materials, in the file SupportingDataGPTtextAnalysis.RData.

XGBoost was used as the classifier throughout this study. Model optimization was performed using leave-one-essay-out cross-validation. In this approach, described in a previous study [11], all the feature vectors from a particular writing sample (with each feature vector representing a paragraph) are left out of the model during training, and the left-out examples are classified based on the entirety of the remaining data. Each paragraph in the data set was then given a probability score of being human or AI, and the overall assignment for each introduction was determined based on the mean probability score received for the set of paragraphs that corresponded to the introduction of interest. Documents with a mean probability of >0.5 were assigned as “human”. After optimization, the final hyperparameter settings used in training and all testing experiments were as follows: use of “binary:logistic” as the objective, num_class = 1, max_depth = 2, eta = 0.4, and 70 as the number of rounds of training.

### 2.1. Testing the Model

After the model was developed and optimized on the 2000 introductions used for training data, multiple test sets representing both human- and AI-generated documents were acquired and classified. For the human documents, ten test sets were generated, one for each journal in the study; each one contained the extracted features from 500 introductions that were written in 2022. Document preparation, feature extraction, and classification were performed using identical conditions to those used in training. The raw data and matrix of features used to classify all these test sets are provided in the Supplementary Materials.

Multiple new AI-generated test sets were produced using GPT-3.5 and GPT-4 so that the performance of the model could be tested on unseen AI-generated data. In each test set, either the title, the abstract, or the full introduction section of a human-written article was provided to ChatGPT, along with instructions to use the material to write an introduction section for a scientific paper. All prompts were different from those used in training, and none of the human-generated “starter text” was from any article that had been used to generate any of the training data. All the prompts used to generate these data sets are provided in Supplemental Table S1, and the actual matrices of extracted features used in testing are found in the following supplemental data file: SupportingDataGPTtextAnalysis.RData. All data processing and machine learning conditions were identical to those used in training.

### 2.2. Detecting ChatGPT in the Wild

The next step of the experiment was to determine the proportion of recently published articles that had leveraged generative AI in the introduction section of the manuscript. Ten new test sets, from the same ten journals used to validate the model, were generated. Each set was constructed from 500 introduction sections of publications from January 2024 (and the preceding months, if necessary). These raw data sets, along with the extracted features used for testing, are provided in the Supplementary Materials. All data processing and machine learning conditions were identical to those used in training.

### 2.3 Validation

To validate the findings, and to obtain more clarity on whether individual journals have different incorporation rates of generative AI, we completed one more experiment. In this case, the two largest journals in the study were selected: *Sensors* and *International Journal of Molecular Sciences*. For each journal, all the introductions, starting with the 1001st published paper for the year 2023 through to the end of the year, were downloaded into a single directory and used, as described above, to build a single feature matrix (test set) representing the original text data. Using the portion of the test set that corresponded to the journal’s last 3000 articles from the end of 2023, we performed supervised classification, as described above, to determine the percentage of articles assigned as AI-written in the “post ChatGPT” era. To find the false positive rate in this data set, we used the portion of the data that corresponded to the first 1000 articles in the data set (which were published in the beginning of 2023) and performed supervised classification, as described above. The overall incorporation of ChatGPT in the late 2023 articles is the total rate (measured from the last 3000 articles in 2023) minus the false positive rate (from 1000 articles from the beginning of 2023). The relevant data matrices are found in the Supplementary Materials in the RData file mentioned previously, and a descriptor of the naming conventions is also found in the Supplementary Materials; it is titled “Supporting Materials Data Explainer”.

## 3. Results

**Figure 1.**
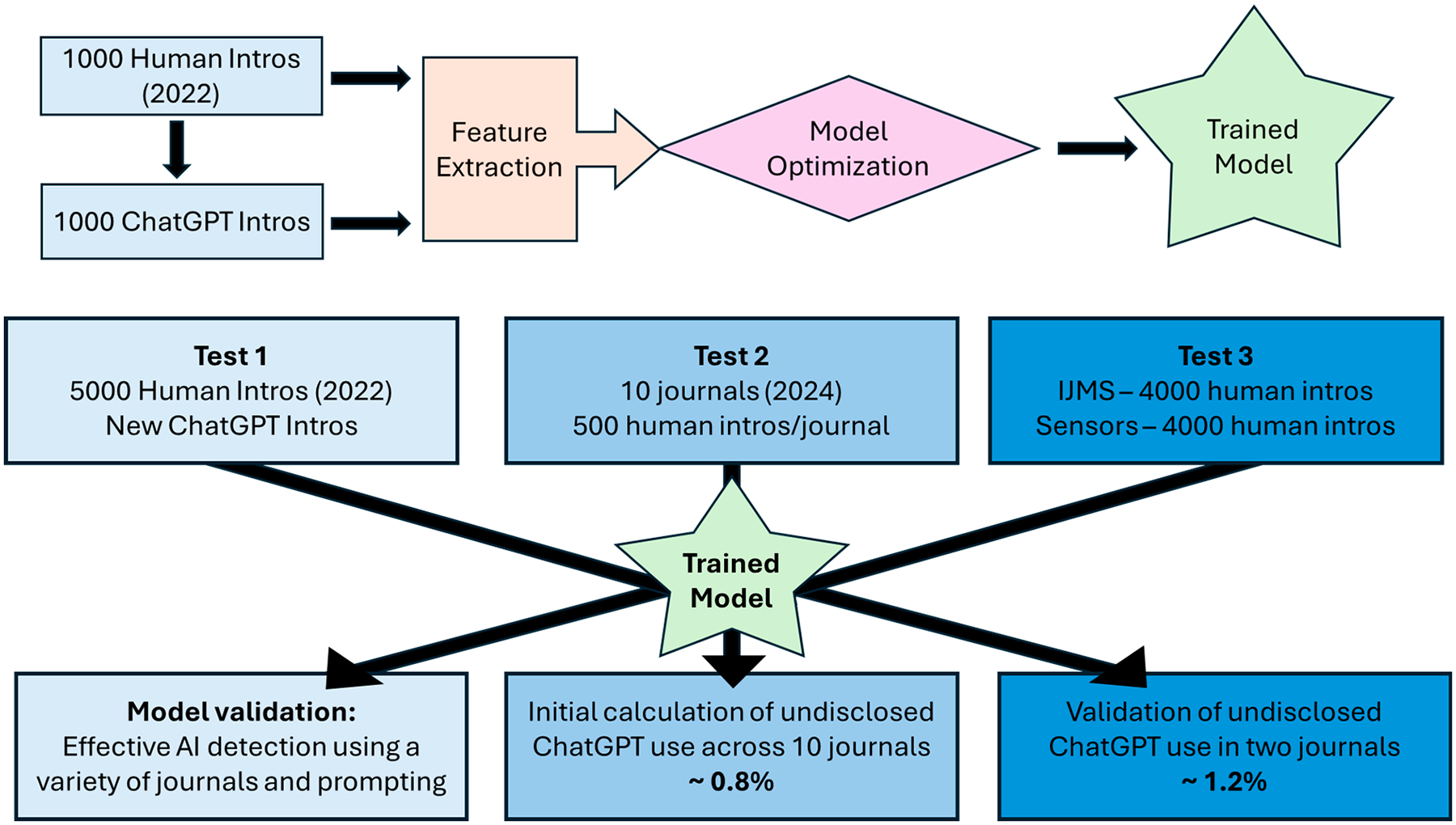
Overview of the study. **Top**: Method development and optimization. **Bottom**: The three tests performed: model validation (test 1); initial rate of ChatGPT usage in ten journals (test 2); secondary validation of ChatGPT usage in two journals (test 3).

The overall workflow of experiments conducted in this study is shown in Figure 1. The first goal was to build an effective supervised classification model to differentiate text that was fully human-written from that which leveraged AI during the writing process. To achieve this goal, a training set of 2000 introductions (half were human-written and half were AI edits of the human-written documents) were generated. A set of 23 features based on stylistic differences was developed, as described in the Experimental Procedures section (Section 2), and used for classification. XGBoost, an off-the-shelf classifier that uses decision trees, was selected as the classifier because it had shown success in similar tasks [11,12]. This approach of using XGBoost and stylistic features is beneficial because it does not require training a neural network or necessitate dimensionality reduction through embeddings, which are common steps in other text classification workflows that typically rely on neural networks. After optimization, described in the Experimental Procedures section (Section 2), the strategy yielded a model with an overall accuracy of 95% using LOOCV. Performance was better for the human-generated documents (99% correct) than the AI-generated ones, which were correctly assigned ~91% of the time. Higher accuracies on the AI-generated documents are easy to obtain at the expense of accuracy on the human-generated documents, but we reasoned that using a strategy that performs best on the human-generated documents would produce less noise in the data, making small changes in the proportion of fully human-generated documents easier to detect.

**Figure 2.**
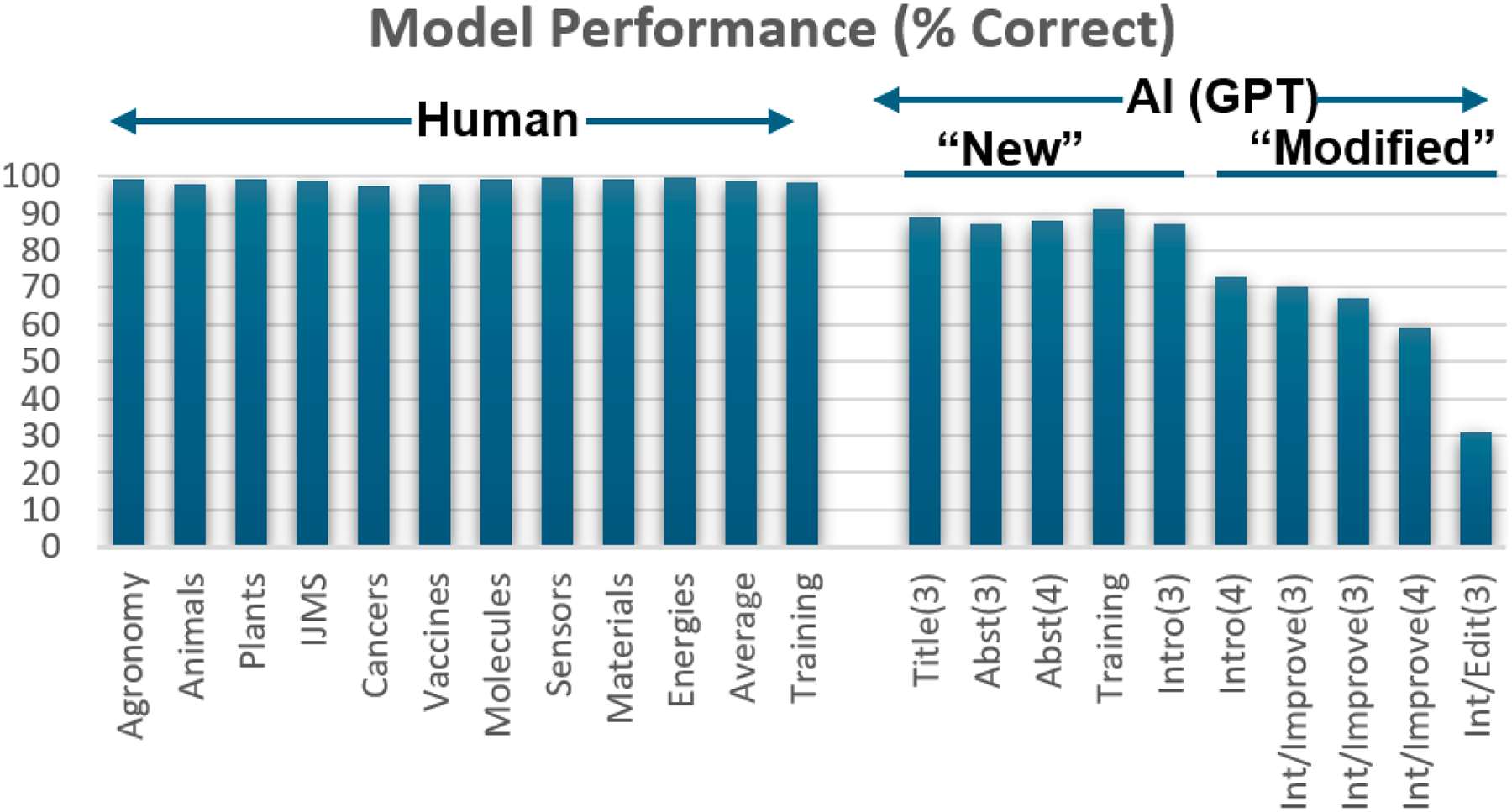
Model performance on diverse data sets including introductions (500 each) from ten MDPI journals (**left**) and ten different data sets generated by ChatGPT (**right**). Each AI-generated data set used prompts that were different from the training prompt, and each contained some human-provided text, which was from a title, abstract, or introduction of a human-written publication. Numbers in parentheses (3 or 4) indicate the version number (GPT-3.5 or GPT-4). All prompts, and more detail about each AI-generated data set, are provided in Supplemental Table S1.

After model development and optimization, the first test (as shown in Figure 1) was a simple assessment of the model on unseen test data, both human-generated and AI-generated. As described above, 500 new documents were used from each of the ten journals in the study, and after feature extraction and supervised classification, the performance of the model was determined and is shown in Figure 2. The developed model was highly effective at accurately assigning the articles as human-written across all ten journals tested, with the overall correct assignments for each journal ranging from 98% to over 99%, and an average percentage of correct assignments of almost 99%. These results closely replicate the performance of the model during the validation (by LOOCV) of the training data. We note that since ~99% of the articles are detected as “human”, the false positive rate of detecting ChatGPT with this model is ~1%. We account for the false positive rate in the model when testing articles from 2024, as described in a later section.

In addition to testing the model on new data written by humans, a second goal of Test 1, shown in Figure 1, was to determine the effectiveness of the model in detecting AI-generated writing in a variety of circumstances. Nine unique sets of AI-generated introductions were used for this purpose; each set had source material and a prompt that was different from what had been used in training; prompts are supplied in Supplemental Table S1. These AI-generated introductions were correctly assigned to varying degrees, depending on the amount of human text provided to ChatGPT and the task requested in the prompt (new writing vs. improving vs. editing grammar). In cases where the introduction was newly written, the detector correctly classified the documents more than 85% of the time, regardless of the quantity of human text provided for inspiration, the prompt, the source of the human text, or the model (GPT 3.5 or GPT 4). See Figure 2. The success rate for detecting AI-generated text decreased, not surprisingly, when the prompts specifically requested less intervention from the AI text generator. For example, when a full introduction was provided but the prompt included instructions to simply “improve” existing text, these introductions were detected as AI-generated less frequently than when the prompt asked for a “new” introduction, even if a fully human-written introduction was provided in both cases. When the instructions “This is an Introduction… Edit the grammar…” were issued, the resulting data sets were detected as AI-generated with low frequency (~30% of the time; Figure 2). The lower detection rates for the “improve” task and the “edit grammar” task are fully expected, as less AI intervention leads to a writing sample that contains more human-written text. In these cases, the documents would be less readily identified as AI-generated by any detector.

The goal of Test 2 (see Figure 1) was to calculate the undisclosed use of ChatGPT in papers written in 2024 across the 10 different journals in the study. Since Test 1 showed the false positive assignment rate for each journal (this is the percentage of articles misclassified as AI in each journal), these values were used in Test 2 as the false positive detection rate in each journal. The true positive rate, then, is the total percentage of AI detected minus the false positive rate, on a journal-by-journal basis. Table 2 shows the total detection rates in the 2024 articles, by journal, and the true positive rate by journal.

The true incorporation rate of ChatGPT, averaged across the ten journals, is only about 1%. We considered whether a detection rate this low could be explained by random chance. When the numbers of articles classified as “AI-generated” are compared between 2022 and 2024 using a paired student’s t test, the increase in AI-generated documents in 2024 is statistically significant. *p*< 0.001; 95% confidence interval is 0.6% to 1.2% AI in 2024.

Because the detection rate from Test 2 is low but statistically significant, we conducted an additional experiment to validate the findings on two different and larger data sets. The two largest journals in this study, *IJMS* and *Sensors*, were selected, and the last 3000 articles that each journal published in 2023 were used as the test set; furthermore, 1000 new articles from these two journals were also used to recalculate a false positive AI detection rate. See Experimental Procedures for more details. Using the same feature extraction step and trained model, the false positive detection rate for each journal was determined to be 1.1% for *IJMS* and 0.4% for *Sensors*. Then, using these data and determining the nominal rates of AI detection at the end of 2023, which were 2.3% and 1.6%, respectively, we calculated the true AI incorporation rate. Both data sets have incorporation rates of 1.2%. See Table 3. These data, drawn from larger sample sizes than those represented in Table 2, validate the earlier overall conclusion that AI usage is detectable in these journals at a very low incorporation rate, and shed new insight on the journal-by-journal variability. Within the larger sample sets, no difference in incorporation is detected between these two journals.

## 4. Discussion

Overall, the results from Test 1 demonstrate that we were able to build an effective model to assess the presence of ChatGPT in introductions from a variety of scientific journals. The model’s performance in accurately identifying human-generated data as being from humans is particularly strong. Its performance in correctly identifying ChatGPT-generated articles is also good but weakens as the amount of intervention requested in the prompt decreases. Considering that both the human-generated and ChatGPT-generated text contain a significant amount of human writing in the first place, the classification task in this challenge is quite daunting, and we expect that the performance obtained herein is likely close to optimal, even with additional training or larger data sets.

The truly new part of this work, compared to previous demonstrations of discriminating between human-generated and ChatGPT-generated scientific articles, is in the application of this tool to actual papers written since the release of ChatGPT. This analysis represents the only quantitative assessment of actual incorporation of ChatGPT into the scientific literature using a method that can identify ChatGPT usage that would evade human detection; it is based on an assessment of 19,000 manuscripts, and it covers a wide range of journals, varying both in their impact and their topical coverage. In two different experiments, Test 2 and Test 3, the incorporation rate of ChatGPT was estimated to be about 1%.

The minimal implementation of ChatGPT in these journals surprised us at first, considering the hype associated with the rollout of large language models. Professional scientists perhaps have not yet embraced this technology in the final stages of manuscript writing because they do not believe the benefits (of somewhat less time spent writing) outweigh the potentially damaging reputational costs, since the use of the technology initially was viewed with suspicion and associated with plagiarism [3]. In an era where deepfakes, disinformation, and distrust shade the public’s assessments of the information it views, we are quite pleased to see that the scientific literature is still written by scientists, and the integrity of mainstream publication venues has not been eroded by undisclosed generative AI.

We note that this study has some limitations in that we cannot distinguish between authors who use generative AI to write the introduction for them and authors who use it to edit an already complete rough draft. We suspect that in most cases, ChatGPT was used mainly for editing, since more scientists view this as an ethical use of AI. If, in fact, all the incidences of ChatGPT usage in the data sets were a result of using it for editing, then we have likely underestimated the true incorporation rate of ChatGPT, since testing on ChatGPT-*edited* introductions indicates that the AI-editing task is detected about 2/3 of the time, whereas the task of generating text anew is typically detected about 80–90% of the time. See Figure 2. With this in mind, if all of the ChatGPT usage was based on late-stage edits, significantly revising a full draft that had all the information provided already, we estimate the true usage rate to be between 1 and 3%, which is still quite low. We note that this value is similar to a survey result which indicated that 4–5% of postdocs (15% of ChatGPT users, which comprises only 31% of the overall group surveyed) said they used ChatGPT for writing manuscripts [21].

Additional studies could be conducted to expand the scope of this work in the future. For example, the study could be expanded to test data that possibly comes from other LLMs, including Gemini, Claude, and LLaMA 2. Also, additional human data could be studied. While ten different journals were tested here, more journals within each field and in more fields overall could be probed, as subtle differences may exist within various subdisciplines. We also note that low-impact journals were not a part of this study, and those concerned about AI’s influence in this domain could use a similar strategy to the one we described here to assess AI incorporation at the lower end of the academic publishing spectrum. Finally, very restricted editing tasks, such as ones with prompts like “make only necessary changes to fix the grammar” are very difficult to detect in this model or by any other approach. Such implementations of generative AI are not a use case that we are interested in detecting, as they pose no risk to the quality of the papers.

Moving forward, journals may want to consider removing their requirements for disclosing generative AI use when it is leveraged as an editing tool. According to a recent survey, almost 20% of postdocs are already leveraging the tool for restricted editing tasks [21]. After almost two years of ChatGPT being widely available, the potential problems with it (including bias [2] and hallucination [22]) are well known, and the overwhelming majority of authors seem to be taking responsibility for the final content of their papers by using this new tool for late-stage edits and not content generation. Our analysis reveals that very few authors are currently using ChatGPT to write their papers for them. Moving forward, research associated with generative AI and scientific publishing should perhaps focus on how to apply the technology in creative ways that save authors time while not compromising on writing quality, accuracy, or research integrity.

## 5. Conclusions

Herein, we developed an effective classifier that can distinguish human-written scientific introductions from those generated or edited by ChatGPT. The method is effective at detecting content from both GPT-3.5 and GPT-4, and the ability to detect AI-generated text decreases, expectedly, when the amount of intervention requested decreases (from writing new text vs. improving existing text vs. making only necessary grammar edits). Using this model, we estimated the undisclosed usage of ChatGPT in 10 different MDPI journals. Across all the journals, the incidence rate of AI-generated text was unexpectedly low, measured at about 1%. Based on these results, we conclude that the scientific literature is not becoming polluted with unchecked AI-generated content; rather, authors are using this tool to a very limited extent and/or primarily for editing tasks. Overall, we conclude that the overwhelming majority of scientists publishing in these 10 MDPI journals are using this new technology responsibly, and ChatGPT is perhaps not yet being leveraged to its fullest potential within the broad scientific community. Journal editors’ requirements to disclose AI use may be driving this trend, and perhaps it is time to reconsider these requirements. Scientists own the responsibility for the content in the papers they publish, whether or not they leverage writing assistance provided by AI, and removing barriers to using these tools may drive scientific progress forward more rapidly.

## Supplementary Material

Supplemental Data

## Figures and Tables

**Table 1.** Journals Selected for this Study.

	Highest Impact Journals				Impact Factor 3.5 to 5			Impact Factor 3 to 3.5		
Journal	*Vaccines*	*IJMS*	*Cancers*	*Molecules*	*Plants*	*Sensors*	*Agronomy*	*Materials*	*Energies*	*Animals*
Impact Factor	7.8	5.6	5.2	4.6	4.5	3.9	3.7	3.4	3.2	3
Size (Rank at MDPI)	50	1	12	6	21	4	23	8	7	18

**Table 2.** Finding undisclosed ChatGPT in 10 journals (500 articles each).

	Agronomy	Animals	Plants	IJMS	Cancers	Vaccines	Molecules	Sensors	Materials	Energies
Total AI in 2022	4	10	3	6	12	11	5	2	3	2
Total AI in 2024	7	10	6	12	20	15	10	4	8	8
True AI in 2024	3	0	3	6	8	4	5	2	5	6
% AI in 2024	0.6	0	0.6	1.2	1.6	0.8	1	0.4	1	1.2

Notes: Total AI in 2022 = # detected as “AI” out of 500. This is the false positive rate for the journal. Total AI in 2024 = # detected as “AI” out of 500 from 2024. This is the total positive rate for the journal. True AI in 2024 = Total AI minus false positive AI. Percentage of AI in 2024 is the “True AI” value (which is out of 500) expressed as a percentage.

**Table 3.** Validation of AI incorporation in larger data sets.

Journal	Total Pos. AI (End 2023) in 3000 Articles	False Pos. AI (Start 2023) (1000 Articles)	True Pos. (Total-False)
*Sensors*	1.6%	0.4%	1.2%
*IJMS*	2.3%	1.1%	1.2%

## Data Availability

The raw data used in this article are provided in the Supplementary Materials section.
