# Supplementary material for: Almost Nobody Is Using ChatGPT to Write Academic Science Papers (Yet)
Source: Big Data Cogn Comput. Author manuscript; Available in PMC 2025 Aug 28. (PMC12382442; doi:10.3390/bdcc8100133)
Supplement: Supplemental Data [file NIHMS2084804-supplement-Supplemental_Data.zip › Supporting Materials Data Explainer.pdf]

Explanations for R objects in the supporting data.

Training data: There are four critical, interrelated object types that relate to the training data. They are: MatTrain4 (where the raw text data are found); FileKeyTr4, which assigns each row in MatTrain4 to its original document number in the training set, and filesTr4, which provides the identity of each document in the training set. An elaborated description is in the next paragraph. Most importantly, the actual training data used, which includes the 23 extracted features, are found in the matrix Train4. The first column (feature) in Train4 corresponds to the class identity for each paragraph of the training data used in the matrix.

More detailed explanation: The original training documents used are appended into a single matrix called MatTrain4. Each row in the matrix is a paragraph of training data. The vector, FileKeyTr4 is a numerical vector that reports the document number for each line in the MatTrain4 matrix. For example, the first three values in FileKeyTr4 are 1, 1, 1. This means that the first three rows in MatTrain4 comprise three unique paragraphs from the first document in the training set. Since the fourth entry in FileKeyTr4 is a 2; this indicates that the fourth row of the matrix, MatTrain4, contains text from the second document in the training set. Note that the length of FileKeyTr4 matches the number of rows of MatTrain4. Each document in the training set is described in more detail in filesTr4. Note that the maximum value in FileKeyTr4, 2000, matches the length of filesTr4. In fact, the 2000th entry in filesTr4, 4Huva22_1164, describes the 2000th document in the training set. It is a document written by a human (Hu), with source text from the journal Vaccines (va) in the year 2022 (22), and the specific training document was number 1164 from 2022 (in Vaccines).

A similar naming strategy has been used for the entirety of the test data. The original text is always in Mat*.*. For example MatMol22 and MatMol24 are the human source test data for the journal, Molecules, from 2022 and 2024 respectively. The two files FileKeyMol22 and FileKeyMol24 contain numeric vectors indicating the numerical document identities for each row in MatMol22 and MatMol24, respectively. These numerical identities can be translated to actual documents using the vectors, filesMol22 and filesMol24. As was done with the training data, the file names, displayed in files*.* follow the convention: Journal AbbreviationYEAR_Article#. So the first article in the 2024 data set for Molecules is found by reading the first entry in filesMol24. That entry reads: Mol24_10; so this indicates that the first article in this data set is the 10th article the journal published in 2024.

Abbreviations used for the human test data include: Vac, Sen, Pla, Mol, Mate, IJMS, Ener, Can, Ani, Agro. These abbreviations correlate to the beginning of each journal name (except IJMS, where the commonly known acronym is used.)

Most importantly, the matrices of test data, containing the extracted features from the textual data, are also present in the R file. They are called TestMat*.* For example, TestMatMol22 contains the feature data from the year 2022 and the journal, Molecules, while TestMatMol24 contains the feature data from 2024 and the journal, Molecules. Note that the TestMat's always have the same number of rows as their corresponding FileKey vector, since each row in the TestMat matrix corresponds to a paragraph identity that is provided in the corresponding FileKey.

Finally, data for the ChatGPT-generated test sets are also provided, also following the same convention, using Mat, FileKey, files, and TestMat as the basic descriptors, and the object names for this group of documents all have the abbreviation, GPT, in them. For the data sets that were acquired using GPT-4, the name will specifically include GPT4, not just GPT.

The prompts for ChatGPT used in this study are found in an excel file (Supplemental Table 1), which is also included in Supporting Materials. In addition to the exact prompt used, the version (either GPT 3.5 or 4) is specified, along with the percent of documents correctly assigned for that training or test set.
